# Nonrecurrent Laryngeal Nerve: Precise Detection by Electrophysiological Nerve Monitoring

**DOI:** 10.7759/cureus.2670

**Published:** 2018-05-22

**Authors:** Gunay Gurleyik, Mehmet Torun, Emin Gurleyik

**Affiliations:** 1 Department of Surgery, Haydarpasa Numune Training and Research Hospital,istanbul; 2 Department of Surgery, Duzce University Medical Faculty

**Keywords:** thyroid, surgery, vagus nerve stimulation, anatomical variation, v1 signal

## Abstract

Complication-free thyroid surgery is mainly based on the motor integrity of the recurrent laryngeal nerve (RLN). The nonrecurrent laryngeal nerve (non-RLN) is a rare anatomical variation that may increase the risk of vocal cord palsy. Early identification and exposure of the non-RLN may minimize injury risk. This case report presents functional detection of the non-RLN by intraoperative neuromonitoring (IONM).

Total thyroidectomy was performed under the guidance of IONM on a patient with bulky multinodular goiter. The first step of IONM is pre-dissection stimulation (V1) of the right vagus nerve (VN). V1 at a standard distal point was negative as indicated by the absence of both a sound signal and wave amplitude. The right VN was then followed proximally and dissected under the guidance of IONM. This dissection established a proximal point creating a positive signal that led us to determine the separation point of the non-RLN. The right non-RLN arising from the proximal VN was identified and fully exposed until laryngeal entry. Its motor integrity was confirmed with post-dissection signals. The left RLN was identified at the usual anatomical position that was fully exposed and preserved during thyroid surgery. Total thyroidectomy was then accomplished without complication. The postoperative period was uneventful. Postoperative laryngoscopy confirmed normal vocal cord function.

The non-RLN is accurately identified by IONM during the early part of the thyroid surgery. The absence of a distal VN signal is predictive of the non-RLN. IONM-guided proximal dissection of the right VN leads to the identification of the non-RLN. The prediction of the non-RLN by the absence of a VN signal during an early stage of surgery may prevent or minimize the risk of nerve injury.

## Introduction

Complication-free thyroid surgery is mainly based on the identification and full exposure of the cervical part of the recurrent laryngeal nerve (RLN). The thyroid surgeon should be familiar with the anatomy of the RLN, including all the anatomical variations. The nonrecurrent laryngeal nerve (non-RLN) is a rare anatomical variation that may increase the risk of vocal cord palsy. Early identification of the non-RLN at the beginning of thyroid surgery may minimize the risk of injury. The preservation of proper nerve activity has paramount importance for safer thyroid surgery. It depends on the integrity of motor function that requires electrophysiological confirmation during surgery by intraoperative nerve monitoring (IONM). It is a widely accepted method to evaluate the motor activity of the RLN during thyroid surgery [1, 2]. The proper and standard use of IONM may help to detect a nonrecurrent course of the inferior laryngeal nerve.

In this paper, we present a case of the non-RLN and electrophysiological detection of a nonrecurrent course of the inferior laryngeal nerve by the first step of standard IONM.

## Case presentation

A 53-year-old woman presented to our department with signs and symptoms of multinodular goiter (MNG). Bulky hypertrophy of the thyroid gland at the anterior neck was determined by inspection. Larger nodules with regular margins were palpated in both lobes by physical examination. Serum thyroid-stimulating hormone and free thyroxine levels were normal by biochemical analysis. Thyroid ultrasound revealed multiple nodules in both lobes. Bigger nodules were a 50 x 36 x 40 mm hypoechoic solid nodule in the right lobe and a 33 x 25 x 37 mm isoechoic solid nodule in the left lobe. Fine needle aspiration from dominant nodules revealed benign cytology. The diagnosis was MNG. Total thyroidectomy under the guidance of IONM was planned as the surgical treatment. Informed consent was obtained from the patient.

**Figure 1:**
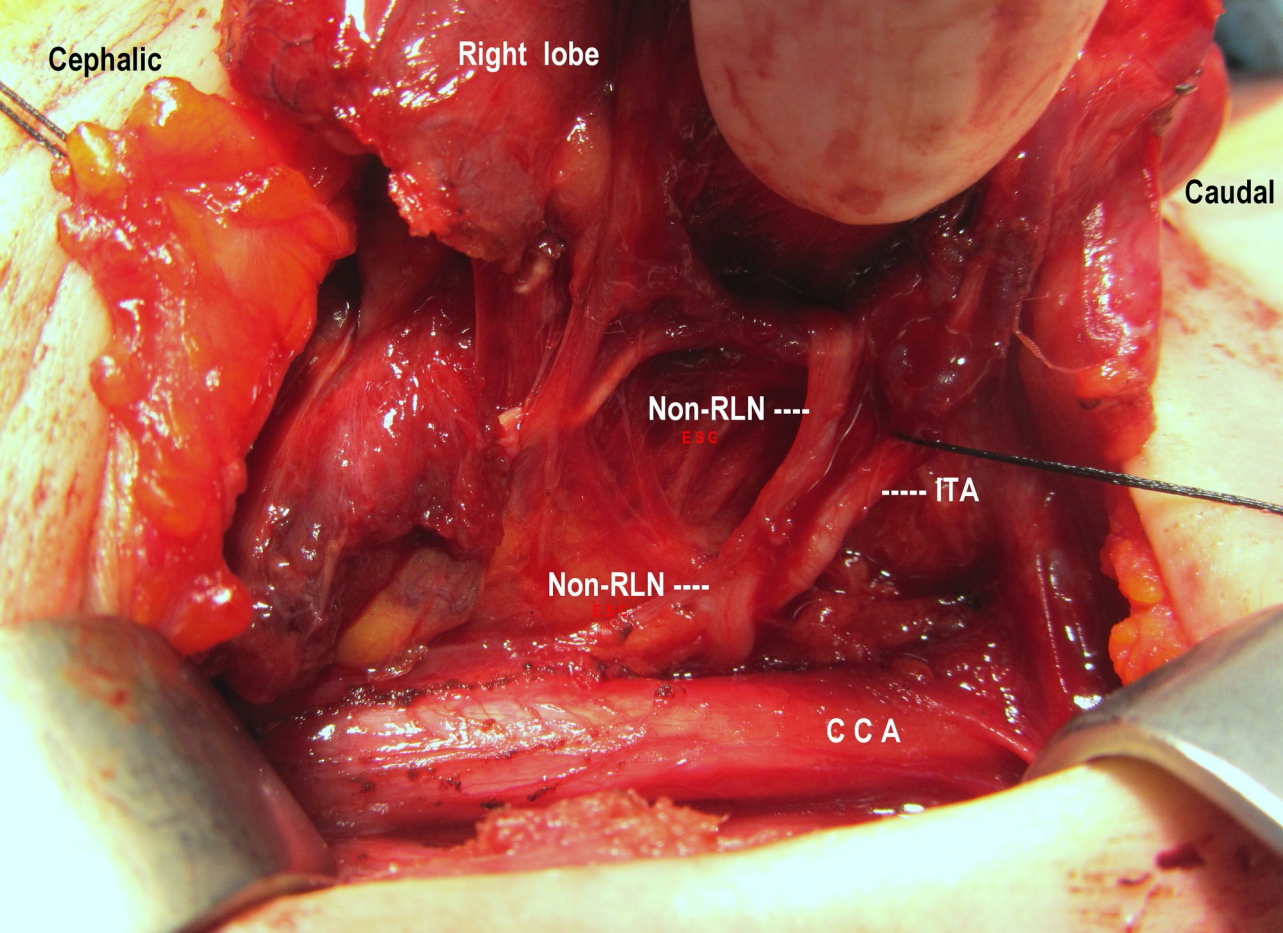
Nonrecurrent laryngeal nerve (non-RLN) Full exposure of the right non-RLN has parallel course with the inferior thyroid artery (ITA) from the posterior to the common carotid artery (CCA) until laryngeal entry.

The right thyroid lobe was partially mobilized after ligation of the middle thyroid vein. The carotid sheath was incised, and the right vagus nerve (VN) was located behind the carotid artery and the jugular vein. Direct stimulation of the VN with the stimulator probe at a standard distal point did not create a sound signal. The absence of a distal V1 (d-V1) signal revealed the early proximal separation of the inferior laryngeal nerve and eventual presence of the non-RLN. The carotid sheath incision was extended toward the cephalic direction. The right VN was proximally followed under the guidance of IONM with serial electrophysiological stimulation to identify the separation point of the inferior laryngeal nerve. A positive signal from a proximal point of stimulation (p-V1 = 648 µV) helped us to locate the separation point of the right inferior laryngeal nerve. Stimulation of the inferior nerve when first identified at the separation point posterior to the carotid artery created a positive sound signal and wave amplitude (R1 = 661 µV). Both lower and upper poles of the right lobe were carefully dissected, and the lobe was mobilized medially. The right non-RLN arising from the proximal VN was identified and fully exposed until laryngeal entry (Figure 1). Vagus signals were negative if the stimulator probe’s contact point was located distal to the non-RLN separation and positive if it was located proximal to the separation. Post-dissection non-RLN (R2 = 721 µV) and proximal VN (p-V2 = 663 µV) signals and wave amplitudes were obtained and recorded after full mobilization of the right lobe. The left lobe was also dissected and mobilized. The left RLN was identified at the usual anatomical position that was both fully exposed and preserved during thyroid surgery. Left V1, R1, R2, and V2 signals and amplitudes were also obtained and recorded. A total thyroidectomy was performed under the guidance of IONM. Both pre- and post-dissection stimulation showed functional integrity of the laryngeal nerves. The postoperative period was uneventful. Postoperative laryngoscopy confirmed normal vocal cord function. The patient was discharged on the second postoperative day. The histopathological diagnosis was follicular nodular disease. The patient is euthyroid with levothyroxine (LT4; 100 µg/day) replacement.

## Discussion

Anatomical variations of the RLN, including the non-RLN, deserve special attention for safer thyroid surgery. Prediction of the presence of the non-RLN preoperatively or at an early stage of surgery can eventually minimize injury risk to the nerve [2-4]. Since July 2011, we have been using IONM in all patients undergoing thyroid surgery. Functional identification by IONM seems to be a useful adjunct to visual identification of the RLN, especially in cases of anatomical variations. Electrophysiological monitoring of the VN theoretically may predict a nonrecurrent course during the early intraoperative period and may facilitate identification of the non-RLN. The present case is a typical example of a prediction of the non-RLN by IONM.

The incidence of the non-RLN is very low and has been reported to be between 0.5% and 3% [2-5]. Preservation of anatomical integrity and motor activity of the nerve has paramount importance for successful thyroid surgery. The utilization of IONM of the RLN in thyroid surgery adds a new dimension to the standard of visual nerve identification since it enables functional nerve testing at the most vulnerable area of the dissection [2]. Nerve monitoring begins with the first step of VN stimulation (V1) at the level of the lower third of the thyroid. The absence of both a signal and an electrophysiological wave amplitude at this level reveals a disorder of signal transmission by motor fibers of the inferior neural system to the intrinsic laryngeal musculature. Taking into account the proximal branching of the laryngeal nerve from the VN as an anatomical variation, the absence of motor function after distal V1 stimulation reveals the presence of the nonrecurrent course of the nerve [6]. In our patient, the absence of electrical conductivity in the motor nerve after the right distal VN stimulation (d-V1) urged us to dissect along the VN proximally, with serial electrophysiological stimulation, until the separation point of the non-RLN. Early dissection was performed in an upward direction along the right VN under the guidance of IONM. A positive signal from a proximal point of stimulation (p-V1) helped us to locate the separation point of the right inferior laryngeal nerve from the VN in our patient. Early separation of the right non-RLN from the VN reveals the absence of the standard V1 signal. Identification of the non-RLN arising from the proximal VN provided safe exposure during thyroid surgery. The VN signal was negative if the stimulator probe contact point was located distal to the non-RLN separation and positive if it was located proximal to the separation. Therefore, a negative right V1 signal at the distal point appeared as an early intraoperative indicator of the non-RLN [6].

Chiang et al. [7] reported that non-RLNs without preoperative recognition were successfully detected at an early stage of surgery by IONM, with a negative response from lower vagal stimulation. Cai et al. [8] reported that the separation point of the non-RLNs was identified and precisely localized with IONM. Gao et al. [9] identified nine non-RLN cases that were also successfully detected at an early stage of surgery using IONM. A negative response from lower vagal stimulation indicated the occurrence of a non-RLN. Donatini et al. [4] reported that using a systematic IONM could increase the detection of the non-RLN and decrease the incidence of nerve palsy in cases of the non-RLN. Kamani et al. [10] identified 10 right-sided non-RLNs by application of IONM. They reported that monitoring vagal stimulation at the defined distal and proximal points provides reliable verification of the presence of the non-RLN. Signals derived from the VN were positive if derived proximal to and negative if derived distal to the branching of the non-RLN [3]. Our results of the present case demonstrate the importance of IONM, and especially right V1 stimulation and signal detection, in predicting the presence of the non-RLN at an early stage of thyroidectomy. Previous reports based on IONM application in non-RLN cases also verify the role of a negative V1 signal in enhancing both the detection and safe identification of the non-RLN [4,6,8-10].

## Conclusions

The absence of an early V1 signal to distal stimulation of the right VN indicates an interruption of the transmission path secondary to the early branching of the inferior laryngeal nerve before the stimulation point. Visual and functional identification of the separation point of the non-RLN is accomplished by proximal dissection under the guidance of IONM. Therefore, IONM verifies the presence of the non-RLN at an early stage of the surgery. This assistance is especially important in cases of anatomical variations of the laryngeal nerves. While anatomical variations are not always predicted preoperatively, we propose routine use of nerve monitoring in all thyroidectomy cases as a useful adjunct to visual exposure of the RLN.
